# Improving the performance of enzymes in hydrolysis of high solids paper pulp derived from MSW

**DOI:** 10.1186/1754-6834-6-107

**Published:** 2013-07-25

**Authors:** Dhivya J Puri, Sonia Heaven, Charles J Banks

**Affiliations:** 1 Faculty of Engineering and the Environment, University of Southampton, Southampton, SO17 1BJ, United Kingdom

**Keywords:** Lignocellulose, Cellulases, Enzyme hydrolysis, High solids hydrolysis, Municipal solid waste (MSW), Bioethanol

## Abstract

**Background:**

The research aimed to improve the overall conversion efficiency of the CTec® family of enzymes by identifying factors that lead to inhibition and seeking methods to overcome these through process modification and manipulation. The starting material was pulp derived from municipal solid waste and processed in an industrial-scale washing plant.

**Results:**

Analysis of the pulp by acid hydrolysis showed a ratio of 55 : 12 : 6 : 24 : 3 of glucan : xylan : araban/galactan/mannan : lignin : ash. At high total solids content (>18.5% TS) single-stage enzyme hydrolysis gave a maximum glucan conversion of 68%. It was found that two-stage hydrolysis could give higher conversion if sugar inhibition was removed by an intermediate fermentation step between hydrolysis stages. This, however, was not as effective as direct removal of the sugar products, including xylose, by washing of the residual pulp at pH 5. This improved the water availability and allowed reactivation of the pulp-bound enzymes. Inhibition of enzyme activity could further be alleviated by replenishment of β-glucosidase which was shown to be removed during the wash step.

**Conclusions:**

The two-stage hydrolysis process developed could give an overall glucan conversion of 88%, with an average glucose concentration close to 8% in 4 days, thus providing an ideal starting point for ethanol fermentation with a likely yield of 4 wt%. This is a significant improvement over a single-step process. This hydrolysis configuration also provides the potential to recover the sugars associated with residual solids which are diluted when washing hydrolysed pulp.

## Background

Production of bioethanol as a transport fuel is predicted to reach 100 billion litres in 2015 [1]. At present the demand is met using first generation bioethanol crops such as sugar cane and corn. In the near future, however, it is envisaged that second generation bioethanol made from lignocellulosic materials will begin to contribute. The conversion of these materials to fermentable sugars has been researched widely over the last few years. The final steps required to make the process economic at a commercial scale are linked to achieving glucose concentrations that match the downstream processing requirements for ethanol extraction. Larsson and Zacchi (1995) showed that costs for continuous distillation of ethanol do not rise significantly once an ethanol concentration above 40 g kg^-1^ (4% wt) has been achieved [2]. This requires an 8% wt sugar solution as a starting point for the fermentation, which for many feedstocks is equivalent to an initial lignocellulosic total solids (TS) content of ~20% [3]. Future large-scale production of ethanol from lignocellulosic biomass will therefore require enzyme hydrolysis at high solids contents, and demand not only high conversion yields but also high sugar concentrations in the hydrolysate.

Meeting these requirements presents some challenges, as hydrolysis at high total solids concentrations can lead to a decrease in substrate conversion, sometimes referred to as the ‘solids effect’ [4-6]. Explanations for this include: insufficient mixing [7]; product inhibition as a result of increasing sugar concentration [8,9]; decreased water availability [10,11]; irreversible binding of adsorbed enzyme to the substrate, including non-productive binding to lignin [6,12]; inhibition of enzyme adsorption [5]; and enzyme denaturation [6]. There may also be other, as yet unidentified, reasons for decreased conversion.

Limitations on mixing due to the high viscosity or nature of the substrate are an important aspect of the ‘solids effect’. One method to overcome these is the use of gravity or tumbling mixing, which has been shown to be a superior strategy compared to shaking at high solids concentrations [7]. An alternative strategy is fed-batch substrate addition which reduces the initial viscosity to allow improved mixing, and may therefore decrease the required processing times [13].

Product inhibition is one of the major limitations in realising the full potential of enzymic hydrolysis, as cellulase-containing enzyme preparations are inhibited by glucose and cellobiose [8,14]. For this reason continuous removal of glucose through simultaneous saccharification and fermentation (SSF) was once thought to be the best way of obtaining reasonable titres of ethanol within relatively short process times. Many researchers today continue to believe that this is the best option [15-17]. As the efficacy of commercial enzymes continues to improve, however, it must be questioned whether it is necessary to forgo optimal hydrolysis conditions, as is invariably the case when SSF is used [18]. Even though SSF can overcome the problem of glucose inhibition it has a further slight drawback, as ethanol can also inhibit the enzyme activity. This has been clearly shown for those cellulases taken from cultures of *Trichoderma Resei* used at 30°C [19,20].

When dealing with concentrated sugar solutions, water availability must also be considered. As product sugars and other soluble compounds are released during hydrolysis they bind water, making it unavailable for the enzyme system. This effect has been demonstrated by replacing glucose with mannose, which does not directly inhibit the cellulase system, but its affinity for water makes this unavailable for the enzymes and hence reduces their performance [10]. It has also been shown that as the soluble content of the hydrolysate increases the effect is to pull water away from the surface of insoluble solids, limiting the activity of the enzymes on that surface with a consequent reduction in hydrolysis yield [11]. One method that has been shown to decrease product inhibition and/or increase water availability is the utilisation of membrane reactor systems which remove monomeric sugars after they are produced [21,22]. Most of the published studies use a low concentration of total solids, however, which is unsuitable for industrial application.

Although enzymes are simply catalysts in the process, and can therefore theoretically be re-used, in practice this may be difficult due to denaturing, inhibition, or irreversible binding to the substrate or other non-targeted materials. The extent to which this occurs depends on the make-up of the enzyme mix. Most commercial enzyme preparations contain a mixture of enzymes and their associated binding domains, to ensure a strong affinity with the substrate and its sub-components [23,24]. Cellulases tend to bind strongly to the substrate and after hydrolysis remain associated with the solid fraction. Weiss et al. (2013) showed that by recycling 85% of the insoluble residual solid with its bound enzymes, plus fresh substrate at 15% TS, the subsequent enzyme requirement could be reduced by 30% [25]. A commercial cellulase enzyme preparation will also contain β-glucosidase which cuts cellobiose and cellotriose into glucose monomers [26]. This enzyme is not bound to the substrate, and will partition into the sugar solution after hydrolysis. Cellulases have been used for up to four rounds of hydrolysis [27] whereas β-glucosidase is known to be less stable over prolonged or multistage reaction periods [28]. All enzymes in a commercial cellulase preparation may non-productively bind to lignin making them unavailable for hydrolysis [12] and they can also be inhibited by the presence of hemicellulosic components [8,29]. Various strategies have been tested to overcome these inhibitive parameters including; pre-treatment to remove lignin and hemicellulose [30-33], addition of compounds which reduce non-productive binding [34,35] and the use of simultaneous saccharification and co-fermentation [36]. A detailed discussion of the enzyme system and its limitations can be found in a recent review by Van Dyk et al. (2012) [24].

If lignocellulosic material is to form the primary substrate for a sugar platform biorefinery then a number of factors need to be considered in relation to the product stream. Although the percentage conversion to sugar is a prime consideration, there is also a requirement to produce a high sugar concentration in the hydrolysate [2]. There must therefore be a trade-off between conversion efficiency, conversion rate, and product concentration. Yang et al. (2010 & 2011) showed that it is possible to obtain a very high substrate conversion (85%) in a period of 24 hours using a 3-stage hydrolysis system with intermediate washing steps [9,37]. The sugar stream arising from each stage, however, contained a maximum sugar content of ~5.5% or 55.5 g L^-1^, which is below the ideal value for further fermentation. Once fermented, a hydrolysate of this strength would contain less than 4% wt ethanol, unless the sugar stream was first concentrated by methods such as multiple-effect evaporation or nanofiltration [38] that would reduce the net energy yield.

One reason for enhancing the performance of enzymes is the cost barrier they present to making cellulosic ethanol a commercial reality. It is estimated that the cost of commercially available enzymes still makes up at least 15% of the total cost of lignocellulosic ethanol production [39]. The current research thus aimed to examine a number of inter-dependent factors that control enzyme efficiency and can be manipulated to improve overall performance and product quality. The overall goal of the work was to maximise the efficiency of enzyme use to obtain a concentrated sugar solution from waste feedstock as a sugar platform for a waste biorefinery, without compromising yields or prolonging process times.

## Results and discussion

### Sugar potential of MSW pulp and control

On acid hydrolysis [40] the pulp derived from MSW yielded 55 : 12 : 6 : 24 : 3 of glucan : xylan : araban/galactan/mannan : lignin : ash respectively, while the control filter paper substrate yielded a 84 : 14 : 2 mix of glucan : xylan : araban/galactan/mannan. The MSW derived paper pulp showed a lignin concentration of 24% when compositional analysis was performed using the standard NREL method [40]. Analysis by the FibreCap method [41] showed, however, that only half of this is lignin of plant origin. The remaining portion is unclassified organic matter which was thought to be inert and did not interfere with enzyme hydrolysis.

### Enzyme dosing and substrate feeding strategy

Preliminary experiments using different enzyme addition strategies were carried out to establish the best procedure for enzyme dosing when using the MSW pulp. In these the enzyme preparation was added either directly or mixed with dilution water at a 1:145 ratio of enzyme : pH 5 water. In both cases the hydrolysis was carried out at a TS concentration of 15% for a 48-hour period. The glucan conversion was equivalent in each case, at 67.3 ± 0.03 for direct addition and 66.8 ± 0.02 when diluted. This result confirms that mixing by tumbling allowed even distribution of the enzyme within the pulp.

A fed-batch experiment, in which the same total amount of solid was added but in equal aliquots at hourly intervals, was also trialled in this study but did not show any major advantage over batch addition as there was no increase in the final hydrolysis conversion, only a slightly faster initial liquefaction rate. There was also no increase in conversion when a split batch configuration was used, with half the substrate and enzymes added 6 hours after the first half. As no advantage could be seen at laboratory scale in using either a fed or split batch mode of operation, all further hydrolysis experiments were carried out in batch mode with mixing by tumbling.

### Two-stage hydrolysis with intermediate fermentation

**Figure 1:**
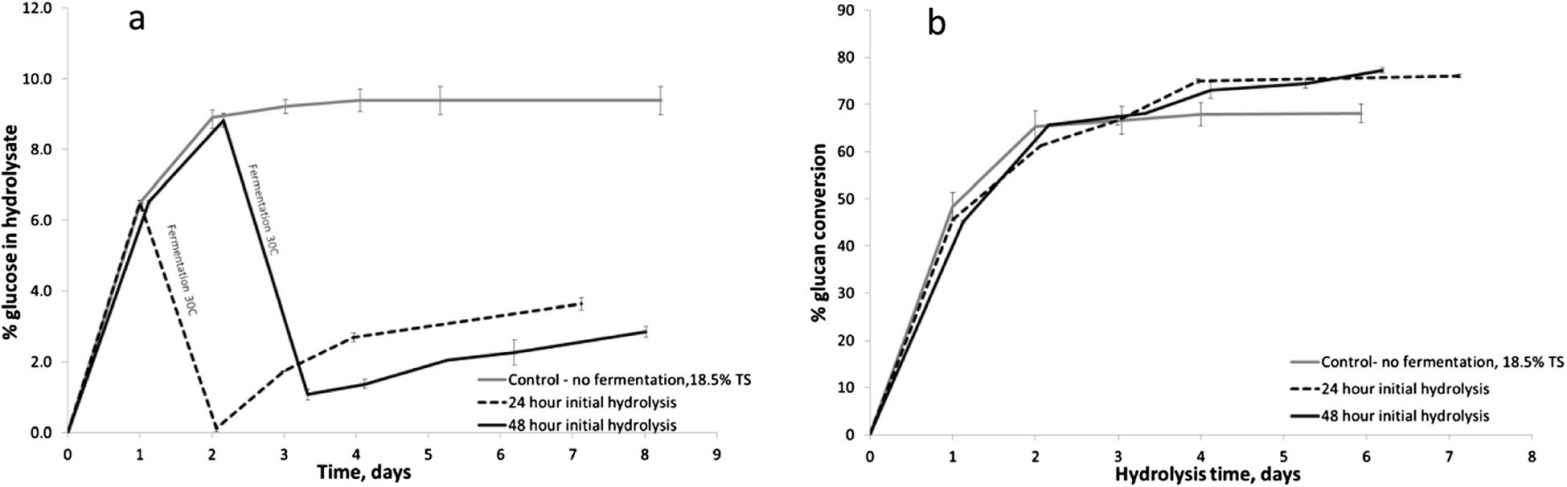
**Evaluation of the effect of an intermediate fermentation step using MSW pulp at 18.5% TS: a) percentage of glucose in hydrolysate, b) overall substrate conversion.** No fermentation (−), fermentation following an initial 24 hour hydrolysis (− −), and fermentation following an initial 48 hour hydrolysis (−).

One aim of this experiment was to assess the effect of changing the length of the hydrolysis cycle and its effects on subsequent glucan conversion. Initial tests were run at 18.5 and 20% TS to determine the time required to achieve a glucose concentration of 8% wt in the hydrolysate. This was 48 hours with 18.5% TS, and 72 hours at 20% TS. The experiment was therefore run at 18.5% TS for 48 hours. The results are shown in Figure 1 and it can be seen that with an initial 48-hour hydrolysis stage the glucose concentration in the hydrolysate was 8.8 ± 0.2% wt. This was then fermented for 24 hours, yielding an ethanol concentration of 2.9 ± 0.6% wt. The yeast was not removed and reaction conditions were then optimised for a secondary hydrolysis (50°C, mixing by tumbling). After a total of 5 days an additional 1.8 ± 0.2% wt of glucose was found in the reaction mixture, giving the potential for a total ethanol yield over the two cycles of ~6 wt%.

In a second experiment the initial hydrolysis cycle was reduced to 24 hours. This gave a lower glucose concentration of around 6.5% wt in the first hydrolysis, which was subsequently converted to 1.8 ± 0.3% wt of ethanol. This was followed by a second hydrolysis stage, which was much faster and yielded around 3% wt additional glucose within 48 hours, rising to ~4% wt over the 6-day hydrolysis period, giving a further 3.7 ± 0.3% wt of ethanol. The total ethanol concentration for the combined two-stage process was 4.2 ± 0.3% wt in a 4-day hydrolysis period and 5.5 ± 0.3% over the total 6-day hydrolysis period. In terms of percentage glucan conversion both two-stage systems gave a higher conversion than was achieved in the 18.5% TS control, as can be seen in Figure. Both the 24 and the 48-hour first stage hydrolysis procedure gave similar final yields, corresponding to total glucan conversions of 76.5 ± 0.5% and 73.1 ± 1.7% respectively (Figure 1b), compared to 68 ± 0.5% in the single-stage hydrolysis control over the same time period. There may be a slight advantage in using the 24-hour initial hydrolysis as early production of ethanol helps to maintain sterility within the system.

The rate of reaction in the second cycle following 24-hour hydrolysis was higher than after the 48-hour initial hydrolysis, and this tends to support the observation made by Pribowo et al. (2012), who suggested that if enzyme recycling is to be undertaken then it should be carried out within 24 hours [42]. The estimated ethanol yield in the second stage of the 48-hour hydrolysis experiment was based only on conversion of the glucose: in all the experiments the hydrolysate also contained up to 1.8% wt xylose, which could potentially be fermented to give additional ethanol if a C5/C6 fermenting strain were used.

The system above works to alleviate product inhibition, but of greater concern is the effect of non-metabolised sugars and other soluble compounds in the hydrolysate/fermentation broth on water availability, as this can also have a negative effect on hydrolysis.

### Two stage hydrolysis with intermediate wash step

When working at high solids concentrations in the hydrolysis stage there is a significant retention of liquid within the residual solids [43]. In a 48-hour hydrolysis test using pulp at an initial 20% TS it was possible to centrifuge the residual solid to 40% TS: the same TS concentration was obtained by centrifugation between 2000–13,000 g. This meant that 65% of the hydrolysate liquid was extractable whilst 35% remained with the solid, representing a considerable amount of unrecovered sugar which may inhibit second stage hydrolysis. To overcome this, after removing the concentrated sugar solution by centrifugation, the residual sugars retained in the solids were removed by washing at pH 5 before being re-suspended in pH 5 phosphoric acid solution for second stage hydrolysis without further enzyme addition.

**Figure 2:**
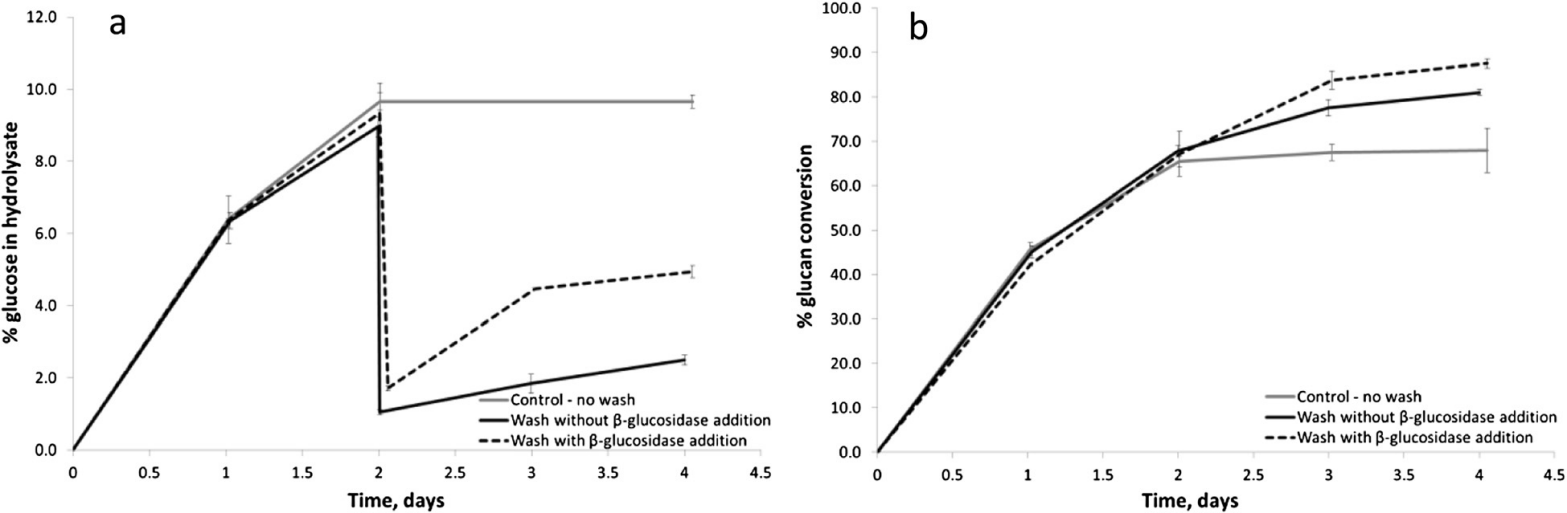
**Effect of intermediate washing and β-glucosidase enzyme addition on MSW pulp (18.5% TS) hydrolysis: a) percentage of glucose in hydrolysate, b) overall substrate conversion.** Unwashed control (−), washed without β-glucosidase addition (−); washed with β-glucosidase addition (− −).

The results showed that when a pulp of 18.5% TS was hydrolysed for 48 hours with 55 mg CTec3 g^-1^ pulp and washed at pH 5, the sugar concentration in the residual solid was reduced from ~9.5% to ~1% (Figure 2a). On second stage hydrolysis of the re-suspended solids a further 14% of glucan could be converted, taking the total conversion efficiency from 67.2 ± 1.3% to 81.2 ± 0.7% (Figure 2b). This effect was thought to be due to an improvement in water availability by removal of the residual sugars, allowing enhanced enzyme activity in the second stage.

### Enzyme recovery through hydrolysate pulp washing

To see whether enzymes as well as sugars were recovered, the washwater was tested for enzyme activity using the filter paper and β-glucosidase activity tests. The tests were carried out with washwaters at pH 5 and 9. The results showed that the cellulase and β-glucosidase activities in the pH 9 washwater were 2.0-3.5 times higher in the washwater at pH 9 than at pH 5. These results are in accordance with those of other researchers who have shown higher enzyme recovery in alkaline conditions [44,45]. It was noted, however, that recovery at the higher pH still only accounted for ≤ 10% of the original FPA enzyme activity. This suggests that most of the cellulase enzyme remains with the pulp and recovery is not feasible. Analysis of the hydrolysate from the secondary hydrolysis showed a slight increase in cellobiose concentration as compared to the initial hydrolysis, indicating a loss of β-glucosidase from the solid faction. This adds support to previous evidence that this enzyme does not bind to the substrate [28]. 15% of the original β-glucosidase activity from the CTec3 preparation was recovered in the pH 5 wash, whereas 30% was recovered in the pH 9 wash. These values do not include any recovered but denatured enzyme, however, which could account for a significant portion of the β-glucosidase initially added [28]. As the cellulase enzymes primarily remain with the pulp, these results indicated that it might be more beneficial to recycle the pulp with its bound enzymes rather than trying to recover enzymes in the washwaters only, as has been the focus in other research [46,47].

### Improving secondary hydrolysis by β-glucosidase addition

The two stage hydrolysis experiment with intermediate wash step indicated that a proportion of the *β-*glucosidase was removed in the washing process. This enzyme is important as it is required to convert cellobiose to glucose and it was thought that the action may release cellobiose from the active sites of cellobiohydrolases, and thus facilitate the processive action of the enzymes [48]. Thus it is an essential component in reactivation of the enzyme systems, and if the depletion observed as a result of washing could be replenished an even higher glucan conversion might be achieved. In this experiment *β-*glucosidase was therefore added as a single enzyme component to the make-up water after the pH 5 wash, at two different concentrations.

When *β-*glucosidase was added at 12.5 mg g^-1^ of original pulp no significant effect was seen, but at 25 mg g^-1^ of original pulp there was a marked increase in glucose concentration giving a hydrolysate with > 5% wt glucose, as shown in Figure 2a. The final glucan conversion following this addition was 88% (Figure 2b). This conversion yield was verified by measuring the weight loss of the substrate which was found to be 87.8%, thus confirming the result.

The final glucose concentration from the two-stage hydrolysis would be ~7.5%, obtained by mixing the concentrated sugar stream (~10%) from the first hydrolysis prior to the washing step with the sugar stream from the second stage hydrolysis (~5%). This is just below the preferred starting point for fermentation, but there is clearly considerable potential for optimisation of the system. Furthermore, this result can be achieved in a 4-day period compared to the 7 days required to obtain a similar yield using the two-stage hydrolysis with fermentation approach.

The experiment was repeated with a second batch of pulp at a slightly higher initial TS concentration of 20 % and a lower initial enzyme dose of 50 mg CTec3 g^-1^ pulp. The same response to β-glucosidase addition was noted; however the final glucose concentration was lower, possibly due to increased solids effects coupled with the slightly reduced enzyme dose. The final sugar concentration of 6% from mixing the primary and secondary hydrolysates was also lower than ideal. This variability clearly shows both the need for optimisation and for careful control of process conditions.

**Figure 3:**
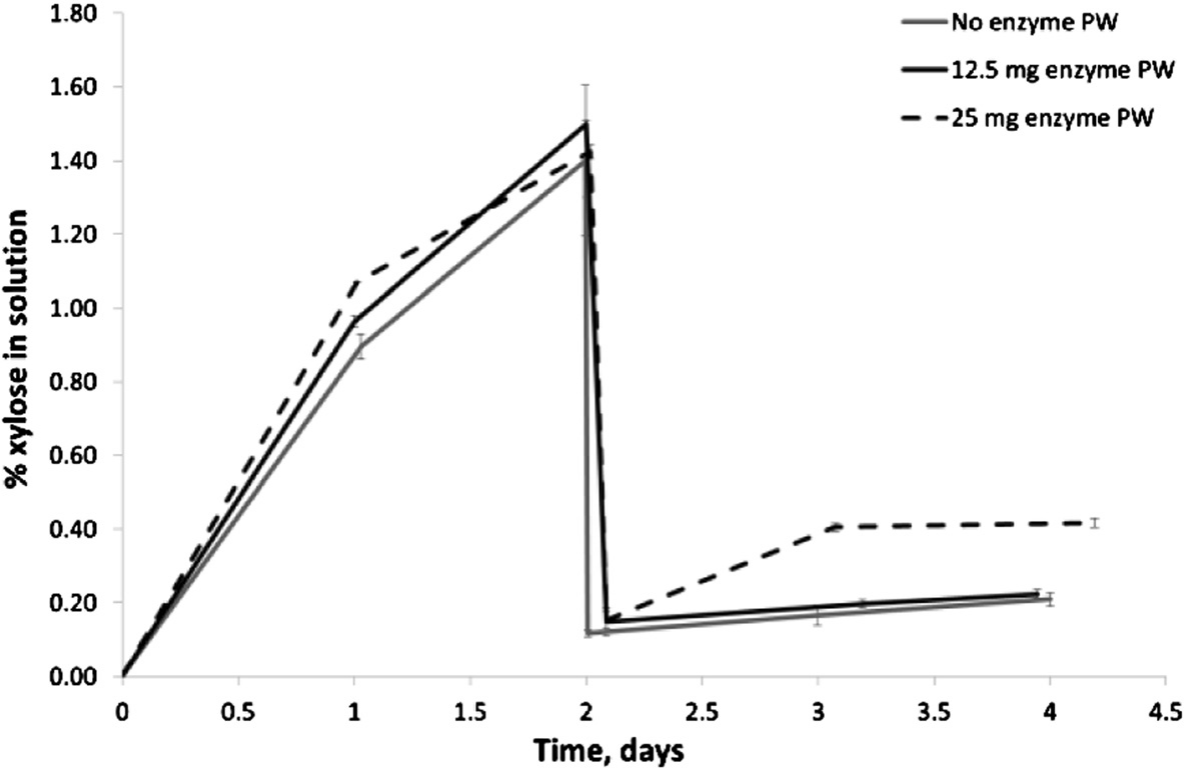
**Effect of different β-glucosidase additions on hydrolysate xylose concentration.** No enzyme post wash [PW] (−), 12.5 mg β-glucosidase g^-1^ original pulp (−), 25 mg β-glucosidase g^-1^ original pulp (− −).

In addition to showing the positive effect of β-glucosidase on glucan conversion, the results also suggest that the enzyme solution may contain some xylanase. As seen in Figure 3, when β-glucosidase was not added or when it was added at a low concentration of 12.5 mg g^-1^ original pulp, the xylose in solution did not increase; whereas when β-glucosidase was added at 25 mg g^-1^ original pulp there was an increase in xylose within the solution which coincided with the increased glucan conversion. Varnai et al. (2010) showed that even a very small amount of xylan, 0.34% on a TS basis, can limit the hydrolysability of a lignocellulosic substrate: adding xylanases to their system gave a 12% increase in cellulose hydrolysis [49]. Other researchers have also shown the detrimental effect of xyloligomers on cellulose hydrolysis and have demonstrated the synergistic effect that can occur when xylanases are added to a cellulase enzyme mix to help increase cellulose conversion [29,33,50]: these studies indicate the importance of the xylan distribution and its role in limiting enzyme hydrolysis.

### Reuse of washwater as a means of reclaiming its sugars and enzyme activity

**Figure 4:**
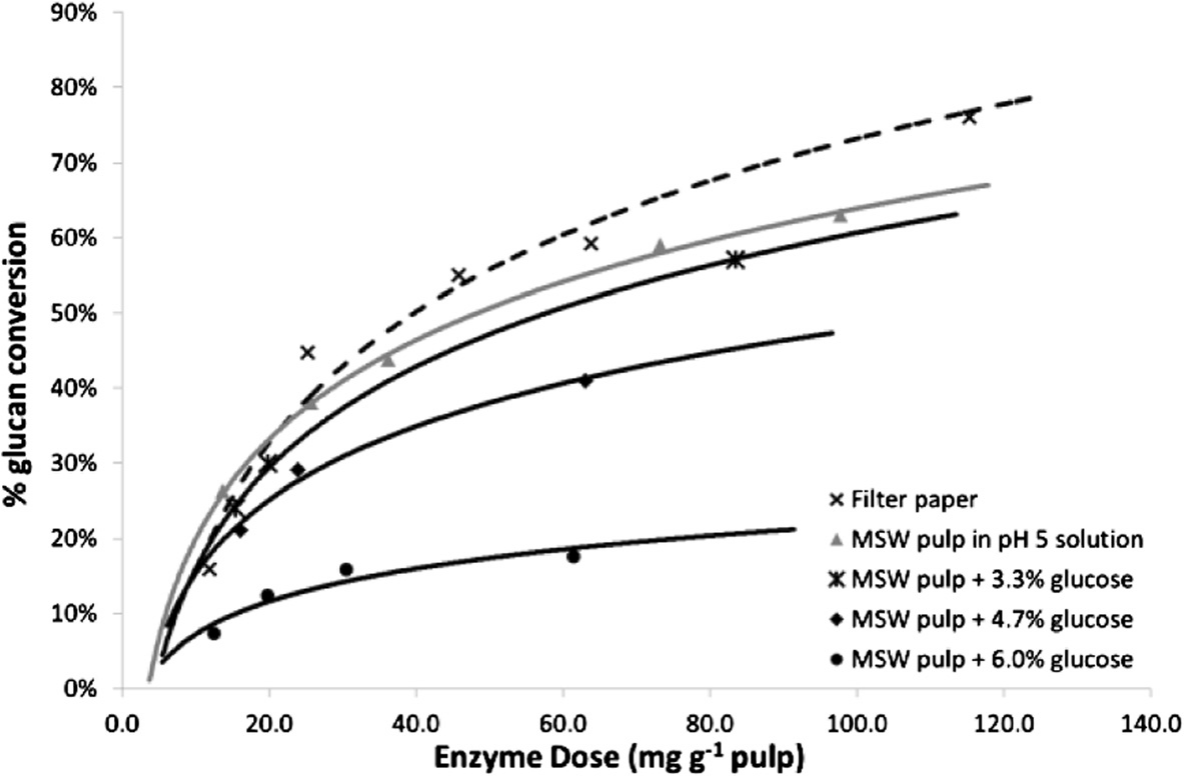
Effect of initial glucose concentration on final glucan conversion over 48 hours.

Hydrolysate pulp washing was necessary as the residual sugars are known to be inhibitory to cellulases; they do however represent a valuable product. It was therefore important to determine the sugar concentration within the pulp at which inhibition occurred. This was tested for Cellic CTec3 using pulp at 6.5% TS spiked with glucose concentrations from 3.3-6% wt. The test was carried out against controls without glucose addition using both pulp and filter paper. The results are shown in Figure 4 and indicate that an initial glucose loading of 3.3% wt did not cause major inhibition over a 48-hour hydrolysis period, regardless of the enzyme dosing used. At 6% wt initial glucose loading, however, significant inhibition was seen. This information was used to establish the minimum amount of water for post hydrolysis washing that would allow re-use of the washwater for dilution of a new batch hydrolysis.

**Figure 5:**
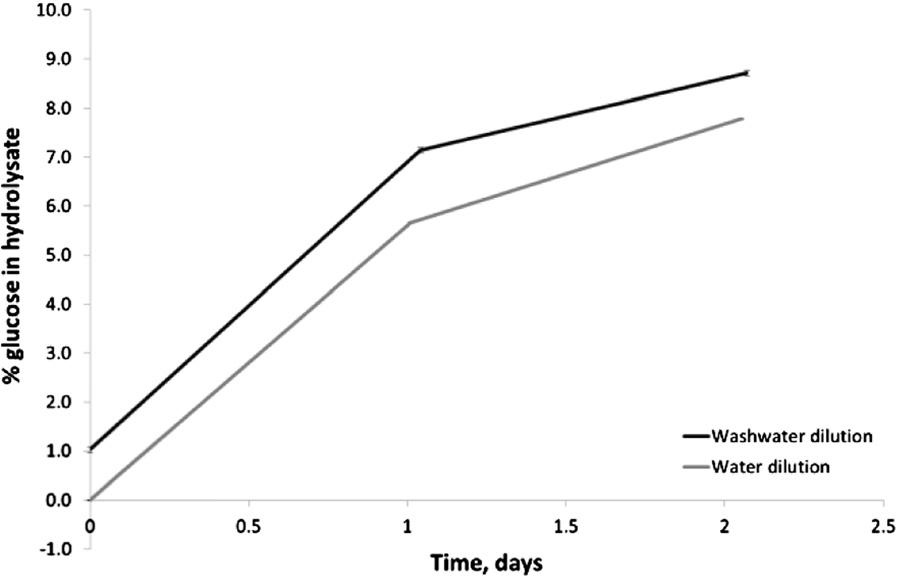
**Effect of the re-use of washwater as dilution water for a new batch of hydrolysis.** Control with dilution by pH 5 water (−), dilution with pH 5 washwater containing residual 1.05% wt glucose (−).

To test this, pH 5 washwater after hydrolysis of pulp at 20% TS was used as part of the dilution medium in preparing a new batch of pulp for hydrolysis, giving an initial glucose concentration of 1.05% wt. It was found that the conversion efficiency over a 48-hour period was the same as with no initial sugar, but the final sugar yield was higher due to the initial glucose content (Figure 5). This approach also ensures that the low but still present enzyme activity in the washwater is utilised.

### Overall discussion

Table 1 compares the 4-day single stage results in terms of glucan conversion with those obtained from both intermediate fermentation and intermediate wash strategies. Two-stage hydrolysis with intermediate fermentation improved glucan yield but this was more successful with a 24-hour than 48-hour initial hydrolysis, indicating that product inhibition was less likely to occur when glucan conversion was more evenly distributed between the two stages. The use of an intermediate wash step that removed residual sugars from the substrate produced an even higher glucan conversion, confirming that water availability may have been a key factor in reducing the activity of enzymes retained on the solid fraction.

**Table 1:** Comparison of all two stage hydrolysis systems after 96 hours of hydrolysis

**Experimental condition**	**% Conversion in primary hydrolysis**	**% Total conversion after secondary hydrolysis**	**% Conversion improvement compared to control**
A. Single stage hydrolysis (96 hours)	68.0 ± 5	-	-
B. Two-stage hydrolysis with intermediate fermentation (24-hour initial hydrolysis)	46.5 ± 0.36	76.5 ± 0.5	8.5 ± 0.5
C. Two-stage hydrolysis with intermediate fermentation (48-hour initial hydrolysis)	65.8 ± 0.3	73.1 ± 1.7	5 ± 1.7
D. Two stage hydrolysis with intermediate wash step	67. 2 ± 1.3	81.2 ± 0.7	13.2 ± 0.7
E. Two stage hydrolysis with intermediate wash step and β-glucosidase addition	67.2 ± 0.8	87.6 ± 0.4	19.6 ± 0.4

The most significant result was obtained when β-glucosidase was replenished after the first stage hydrolysis as this apparently helped to reactivate cellulases that were bound to the substrate. Using this strategy gave a higher glucan conversion at a lower enzyme dose than in many other high solids hydrolysis studies [51-53]. The importance of obtaining a concentrated sugar solution from this high conversion yield should also be noted. The liquid hydrolysate stream from an intermediate wash strategy may not require a concentration step prior to fermentation, saving on energy costs and allowing higher net ethanol yields per tonne of substrate. Moving away from SSF to a separate hydrolysis process also opens up opportunities for use of the concentrated sugar stream for other biorefinery applications beside ethanol production [54].

## Conclusions

Sugar product inhibition could be alleviated using two-stage hydrolysis with an intermediate fermentation step where both hydrolysis and fermentation were performed under their optimum process conditions. Carrying out a 24-hour fermentation step after an initial 48-hour hydrolysis and then readjusting the system for a further hydrolysis step gave a 9% increase in glucan conversion, as compared to a control that did not undergo fermentation.

Washing the residual solids at pH 5 to remove sugars allowed the solid-bound enzymes to show further activity, giving a potential 14% increase in conversion compared to a control without washing. This was attributed to increasing the water availability in the system, by removing xylose and other soluble compounds.

Replenishment of β-glucosidase lost in the washing process further boosted conversion, resulting in a glucan yield of ~88%. Mixing the hydrolysates from the primary and secondary hydrolysis (before and after the wash) gave a glucose solution of just less than 8% wt, the minimum starting point for fermentation, after only 4 days. Furthermore this strategy also allowed the washwater to be re-used for dilution of a new batch of material for hydrolysis. It was found that a glucose concentration of < 3% wt did not cause significant product inhibition of Cellic CTec3. Thus returning glucose at a concentration of 1-2% into a new batch hydrolysis augmented the glucose concentration without affecting the rate of reaction.

## Methods

### Substrate

#### Municipal solid waste pulp

This was provided by Fiberight Ltd from its pilot plant in Lawrenceville, Virginia, USA. The municipal solid waste (MSW) had been first autoclaved and then washed to remove plastic, metals and mineral contaminants, and was supplied at a total solids (TS) content of 30.0 - 37.5% after mechanical dewatering.

#### Control substrate

Fisher Brand filter paper (Cat no. FB59035, Fisher scientific, Loughborough, UK), was used as a defined source of paper cellulose without lignin and ash.

### Enzymic hydrolysis

#### Enzyme, reaction mixture and conditions

The commercial cellulase mixture Cellic CTec3 (Novozymes, Copenhagen, Denmark) was used for hydrolysis. The reaction medium was water adjusted to pH 5 with phosphoric acid. Hydrolysis was carried out in Nalgene™ PPCO centrifuge bottles to which the substrate was added at a concentration of 18.5 or 20% TS before being autoclaved at 121°C for 15 minutes. Once cooled the substrate was charged with Cellic CTec3 enzyme at a concentration of 50–55 mg per g of substrate, equivalent to 6 – 6.5 FPU g^-1^ dry substrate. The mix was then incubated at 50°C in a tumbler mixer at 33 rpm. Hydrolysis was allowed to proceed for periods of up to 8 days, with 0.2 ml samples being taken at regular intervals after centrifugation at 5000 g for 5 minutes. The sugar content of the samples was determined by high performance anion exchange chromatography with pulsed amperometric detection (HPAEC-PAD).

#### Enzyme assays

Filter paper activity (FPA), and β-glucosidase activity were measured using methods adapted from that of Ghose (1987) [55].

Filter paper activity was used to determine cellulase activity of recovered enzymes from hydrolysate after separation by ultrafiltration. Whatman No. 1 filter paper was cut into strips weighing 50 mg. A strip was placed into a test tube with 1 ml of water adjusted to pH 5 with phosphoric acid and equilibrated to 50°C. 0.5 ml of the ultrafiltered test solution was then added, and the mixture was incubated at the same temperature for 1 hour. Further reaction was stopped by placing the tubes in boiling water for 5 minutes, and the glucose concentration was measured by HPAEC. All assays were carried out in triplicate..

β-glucosidase activity was measured by adding 1 ml of a 19.6 mmol L^-1^ cellobiose solution at to 1 ml of ultrafiltered enzyme solution, both at 50°C. The mixture was incubated in test tubes at 50°C for 1 hour. The reaction was quenched by placing the tubes in boiling water for 5 minutes. The glucose released was measured by HPAEC. All assays were performed in triplicate.

The tests were standardised by comparison to the FPA and β-glucosidase activity of CTec3 at a dilution of 1 g enzyme preparation per 80 g of pH 5 water, as this was the enzyme concentration used in this study. Cellic Ctec3 had an FPU of 120 ml^-1^. As the protein concentration could not be determined in the ultrafiltered solution the β-glucosidase activity of the recovered enzyme was compared to that of the fresh enzyme which could produce 8.4 mg glucose per minute per g of enzyme preparation.

### Sugar analysis

#### Sugar potential and MSW compositional analysis

The sugar composition of the MSW pulp was determined using the NREL method for ‘Compositional analysis of structural carbohydrates’ [40]. 0.3 g of pulp was hydrolysed for 1 hour at 30°C with 4.92 g of 72% sulphuric acid. After this the sulphuric acid was diluted to 4% with deionised water, and the mixture placed in an autoclave for 1 hour at 121°C. The residual solids were filtered from the solution, which was analysed for sugar content using HPAEC. The residual solid was dried overnight at 105°C and the dry weight was taken as the lignin and ash content. The solid was then placed in a furnace at 550°C for 2 hours to determine the ash content, and the lignin component was taken to be the total solid minus the ash.

*HPAEC - PAD.* Samples for sugars determination were placed on ice as soon as they were taken, and if not analysed immediately were frozen. Before analysis both fresh and defrosted samples were centrifuged at 13,000 g for 7 minutes in a Galaxy 16DH centrifuge (VWR, UK). The supernatant was diluted and placed in a 5 ml sample vial with a 0.45 μm nylon filter cap. Sugar analysis was carried out on a Dionex DX-500 system using a method adapted from that of Davis (2008) [56]. In this glucose, xylose, galactose, arabinose, mannose and cellobiose were separated at 30°C on a CarboPac PA1 column (250 × 4 mm) in combination with a CarboPac guard column (25 × 4 mm) (Dionex, Sunnyvale, CA, USA). The mobile phase components were 200 mmol L^-1^ sodium hydroxide (A), distilled water (B) and 170 mmol L^-1^ sodium acetate in 200 mmol L^-1^ sodium hydroxide (C). The system set up used a 2.5 μL sample loop and 300 mmol L^-1^ sodium hydroxide post-column eluent at a pressure of 2.76 bar to aid sugar detection.

### Separation of enzyme from sugar solution

#### Ultrafiltration

this was carried out using an AKTA crossflow automated filtration system (GE Heathcare Life Sciences, Uppsala, Sweden). Samples were first filtered through a mesh with a pore diameter of 0.5 mm. The filtered liquid was then run through a 500 kD hollow fibre with a 1 mm lumen i.d. The flow was shear controlled at 8000 s^-1^ with a trans membrane pressure (TMP) of 0.5 bar. The permeate product was collected and used as the feed for a Kvickstart cassette with a 10 kD cut off and flux controlled at a TMP of 1 bar to separate the enzymes from the sugars.

### Fermentation

All ethanol fermentations used Youngs’ super wine yeast compound (Youngs, Bilston, UK). 1 g of yeast was cultured for 24–72 hours in 200 ml of pH 5 basal medium containing 30 g L^-1^ glucose and 10 g L^-1^ yeast extract. The yeast was harvested by centrifuging a portion of the fermentation broth. Once centrifuged the supernatant was removed, leaving a wet yeast pellet of 1.7 g which was re-suspended in 2 ml of pH 5 solution. This suspension was then added to centrifuge bottles containing hydrolysate, following a nitrogen purge. Fermentation was carried out over 24 hours at 30°C with orbital shaking.

### Experimental design to test performance improvements resulting from interventions or changes in conditions

#### Two-stage hydrolysis with intermediate fermentation

Pulp was hydrolysed for 24 or 48 hours followed by a 24-hour fermentation that was sufficient to consume most of the sugar produced. After fermentation, the reaction medium was readjusted to pH 5 to give optimum conditions for hydrolysis, and incubated with tumbling at 50°C for a further period of between 2 and 5 days. During all stages samples were taken for quantification of the type and yield of individual sugars, from which ethanol yield could be calculated. The experiment was carried out at pulp concentrations of 18.5 and 20% TS.

#### Two stage hydrolysis with intermediate wash step

After primary hydrolysis of MSW pulp for 48 hours the hydrolysate was centrifuged and the supernatant, consisting of the concentrated sugar solution, was separated from the residual solid. The residual centrifuged pulp was then washed once for 1 hour with 100 ml of water at either pH 5 or pH 9. The enzyme activity of the supernatants was determined by first separating the enzymes from the sugars by ultrafiltration and then by assaying the filter paper and β-glucosidase activity.

The washed residue was re-suspended in a pH 5 solution (volume equivalent to the removed sugar solution) and, without the addition of further enzyme, was then incubated at 50°C and tumbled to promote second stage hydrolysis. Samples were removed from the hydrolysis reaction mixture at regular intervals and assayed for their sugar concentration using HPAEC.

#### Reuse of washwater as a means of reclaiming its sugars and enzyme activity

The washwater from the enzyme recovery experiment above was used as dilution water for a fresh batch of hydrolysis in which the pulp was autoclaved for 15 minutes at 121°C at a TS concentration of 30–37.5% and then diluted to 20% TS using the washwater.

#### β-glucosidase addition

After centrifugation, washing and re-suspension of hydro-lysed pulp in pH 5 solution, β-glucosidase was added at concentrations of 25 and 12.5 mg enzyme per g pulp (original weight) and hydrolysis conditions were restored. Samples were then taken for sugar analysis over the second hydrolysis period of up to 4 days.

All experiments were carried out in duplicate and repeated twice.

## Abbreviations

FPA: Filter paper activity; HPAEC-PAD: High performance anion exchange chromatography with pulsed amperometric detection; MSW: Municipal solid waste; PW: Post wash; SSF: Simultaneous saccharification and fermentation; TMP: Trans-membrane pressure; TS: Total solids

## Competing interests

The authors declare that they have no competing interests.

## Authors’ contributions

DJP designed and performed the experiments and analysed the data. All authors discussed the results and implications and commented on the manuscript at all stages. CJB and SH contributed to interpretation, supervision and critical review. All authors contributed to the writing of the manuscript, and read and approved the final manuscript.
